# Comparison of Intravenous Medetomidine and Medetomidine/Ketamine for Immobilization of Free-Ranging Variable Flying Foxes (*Pteropus hypomelanus*)

**DOI:** 10.1371/journal.pone.0025361

**Published:** 2011-10-31

**Authors:** Jonathan H. Epstein, Jennifer A. Zambriski, Melinda K. Rostal, Darryl J. Heard, Peter Daszak

**Affiliations:** 1 EcoHealth Alliance, New York, New York, United States of America; 2 Department of Animal Science, Cornell University, Ithaca, New York, United States of America; 3 Department of Small Animal Clinical Sciences, College of Veterinary Medicine, University of Florida, Gainesville, Florida, United States of America; University of Georgia, United States of America

## Abstract

Medetomidine (0.03 mg/kg) and medetomidine/ketamine (0.05/5.0 and 0.025/2.5 mg/kg), administered by intravenous injection, were evaluated for short-term immobilization of wild-caught variable flying foxes (*Pteropus hypomelanus*). Medetomidine alone produced incomplete chemical restraint and a stressful, prolonged induction. Both ketamine/medetomidine doses produced a smooth induction and complete immobilization. The combined medetomidine/ketamine dose of 0.025/2.5 mg/kg produced a rapid induction (232±224 sec) with minimal struggling and vocalization, a complete and effective immobilization period, and tended to lead to a faster and better quality recovery than medetomidine alone or a higher dose of medetomidine and ketamine (0.05/5.0 mg/kg), thus reducing holding time and permitting an earlier release of the bat back into the wild.

## Introduction

The variable flying fox, *Pteropus hypomelanus*, is an old world fruit bat and member of a family Pteropididae, which includes many threatened and endangered species [1], [2]. Flying foxes (*Pteropus spp.*) have been identified as reservoirs for several important zoonotic diseases including Hendra virus (HeV), Australian bat lyssavirus (ABLV), and Nipah virus (NiV) [3], [4], [5]. Disease surveillance in flying foxes has consequently increased in response to public health and economic concerns. Non-destructive epidemiologic studies of individual pteropid bats require the safe collection of biological samples from the bat, which frequently include blood and swabs from the throat, urethra, and rectum [3], [6], [7], [8]. Chemical restraint minimizes the animal's stress and protects the researcher during sample collection [9]. A limited number of injectable anesthetic protocols have been described for pteropid bats. A combination of acepromazine (1.1 mg/kg) and ketamine (11 mg/kg) has been used to restrain the Indian flying fox (*P. giganteus*) [10], xylazine/ketamine (2/10 mg/kg IM) and medetomidine/ketamine (0.03/3–0.06/6 mg/kg IM) has been used in captive variable flying foxes (*P. hypomelanus*) [11], [12]. Likewise, few injectable protocols have been described in free-ranging pteropid bats [10], [11], [12], [13], [14].

Optimal anesthesia *in situ* includes rapid, smooth induction with a complete immobilization period that is sufficient in duration and quality for sample collection; provides a smooth, rapid and uneventful recovery, and ultimately, the safe release of the bat back to the environment. The purpose of this study was to test anesthetic protocols that had previously been used on captive *P. hympomelanus* [12] on wild bats, and assess the efficacy of medetomidine alone and medetomidine/ketamine combinations as chemical restraint agents for the purpose of minimally invasive clinical sample collection. This report describes the use of medetomidine and medetomidine/ketamine for the short-term field immobilization of free-ranging variable flying foxes.

## Materials and Methods

### Animals

*Ethics Statement:* The methods used in this project (described below) were approved by Wildlife Trust's IACUC, #04-08.

Fifty-two variable flying foxes (*Pteropus hypomelanus*) were captured as part of an epidemiologic study of Nipah virus. The bats were anesthetized and blood, throat, urogenital, and rectal swabs; and wing punch biopsies were collected from each bat. Four juveniles were not anesthetized, as they were docile enough to restrain manually. Additionally, 3 bats (07130435 and 07130413 and 07130433) were excluded from the analysis for the following reasons: one had no weight recorded, one was given an initial dose that was not consistent with the protocol and one was not given IV anesthetic. One bat (7140142) was included in the anesthesia failure test, but not in the induction time test because induction time was not recorded. Health status was assessed based on physical exam and a body condition score and an age-class was assigned (juvenile or adult) based on secondary sexual characteristics and dental wear [15].

### Drugs

Commercial preparations of medetomidine HCl (1 mg/ml, Dormitor, Pfizer, Pfizer Animal Health, New York, New York 10017) and ketamine HCl (100 mg/ml; Ketaset, Fort Dodge Laboratories Inc., Fort Dodge, Iowa 50501, USA) were used. Atipamezole HCl (5 mg/ml, Antisedan, Pfizer, Pfizer Animal Health, New York, New York 10017) was used to reverse medetomidine in twenty-nine individuals. Bats receiving medetomidine alone were induced with a dose based on recommendations for rats and rodents (0.3 mg/kg IM) [16]. Bats received initial doses between 0.05 mg and 0.45 mg of medetomidine. The bats from this group that remained immobilized for the entire sampling procedure (n = 4) received an equivalent volume of atipamezole as a reversal agent. Equal volumes of medetomidine and ketamine were combined within three hours of use to make the following concentration: 0.5 mg/ml medetomidine and 50 mg/ml ketamine. This concentration of medetomidine and ketamine was used to give one of two doses: either 0.5 mg/kg medetomidine and 5 mg/kg ketamine or half of that dose 0.25 mg/kg medetomidine and 2.5 mg/kg ketamine. The bats from this group that remained immobilized for the entire sampling procedure (n = 25) received a dose of atipamezole equivalent to half of the total medetomidine volume administered. The anesthetic doses were administered intravenously (IV) in the cephalic (“patagial”) vein located along the cranial edge of the wing membrane between the shoulder and first digit. Atipamezole was administered intramuscularly (IM) in the pectoral muscle. All injections were given with a 1 ml syringe and a 1 inch 22 g needle.

### Experimental protocol

All bats were caught on Tioman Island, Malaysia (2°50′40.56″N, 104°9′34.68″E) using mist nets between 0400 hr and 0730 hr and again between 1800 hr and 2100 hr [17]. Bats were removed from the net and placed separately into pre-weighed drawstring cotton pillowcases that were suspended from tree branches or a rope strung between two trees. During the day, the pillowcases were hung in the shade. All bats were weighed in the pillowcase using a digital fishing scale accurate to 0.01 g (Rapala, USA, www.rapala.com). Of the 45 bats in the study, eleven received medetomidine alone, 18 received the medetomidine/ketamine dose of 0.05/5.0 mg/kg, and 16 bats received the medetomidine/ketamine dose of 0.025/2.5 mg/kg. After injection, each bat was manually restrained with covered eyes until the animal was anesthetized. Heart rate, respiratory rate, and body temperature were recorded at the time of injection and every five minutes until the end of immobilization. The withdrawal reflex was assessed by manually pinching a pedal distal phalanx, palpebral reflex by touching the medial canthus of the eye, and biting reflex was assessed by placing a metal forceps at the commissure of the lips. Induction time was defined as the time elapsed from injection of anesthetic to the loss of withdrawal, palpebral, and biting reflexes. Recovery time was defined as the time elapsed from the injection of atipamezole to the time the animal had regained all of its reflexes. Animals that did not receive atipamezole were not included in the recovery time assessment. Animals that did not become anesthetized after receiving one or two doses of their drug protocol were considered an anesthetic failure event. Following recovery, the bats were offered mango juice orally via syringe to provide additional fluids and energy, and then were released at the site of capture.

### Statistical analysis

A chi-square test was used to compare anesthetic failure rate of the three protocols. Based on the initial results, the two successful medetomidine/ketamine protocols were compared using a chi-square test to assess whether one protocol required significantly more doses of anesthetic. The mean induction and recovery times were calculated ± the standard deviation and were compared using the Mann Whitney U test. A Fisher's exact test was used when sample sizes were too low (less than five observations per cell) for a chi-square. A p-value of <0.05 was considered significant.

## Results

Forty-five animals were included in the analysis (seven juvenile and 14 adult males; three juvenile and 21 adult females). Medetomidine alone produced an adequate plane of anesthesia in 55% (6/11) animals, while medetomidine/ketamine doses of 0.05/5.0 mg/kg and medetomidine/ketamine doses of 0.25/2.5 mg/kg produced anesthesia in 100% of animals (18/18 and 16/16 respectively). Medetomidine alone was significantly more likely to fail than either medetomidine/ketamine protocol at producing anesthesia (Pearson χ^2^ coefficient: 17.4, p<.001).

There was no significant difference in induction time between the two medetomidine/ketamine doses (U = 91, p = 0.12) nor the number of bats requiring multiple doses to induce anesthesia (χ^2^ coefficient: 2.6, p = 0.13). However, it is likely that with larger sample sizes a significant difference will be observed as the 0.5/5.0 mg/kg medetomidine/ketamine dose group had a mean induction time of 156 seconds and 1/18 (5.6%) of animals needed an additional dose of anesthesia and the 0.25/2.5 mg/kg medetomidine/ketamine dose group had a mean induction time of 232 seconds and 4/16 animals (25%) required additional doses to induce complete anesthesia. There was a trend that bats receiving the 0.25/2.5 mg/kg medetomidine/ketamine dose recovered faster (384 sec) than those receiving the 0.5/5 mg/kg medetomidine/ketamine dose (759 sec) but this was not significant (U = 29.5, p = 0.76). The above results are summarized in Table 1.

**Table 1:** Percent immobilized, induction time (mean ± standard deviation) and recovery time (mean ± standard deviation) for wild-caught variable flying foxes (*Pteropus hypomelanus*) administered medetomidine and medetomidine/ketamine by intravenous injection.

Drug Regimen	Range (mg/kg)	n	% immobilized	Induction (sec)	Recovery (sec)
Medetomidine 0.5 mg/kg	0.15–0.45	11	55% (6/11)*	NA	NA
Medetomidine/ketamine 0.25/2.5 mg/kg	0.022/2.2–0.028/2.7	16	100% (16/16)	232±224	384±257
Medetomidine/ketamine 0.05/5 mg/kg	0.045/4.5–0.064/6.4	18	100% (18/18)	156±156	759±759

*Fisher's Exact test; p<0.001.

The use of medetomidine alone resulted in a significantly higher anesthetic failure rate compared to the other two anesthetic protocols.

## Discussion

Medetomidine is an attractive injectable chemical immobilization agent when inhalant anesthesia is not available because of its portability, stability at tropical temperatures, wide therapeutic index (in dogs) and ability to be rapidly and completely reversed by atipamezole [16]. It has been used either alone or in combination as an immobilization agent in other domestic or wildlife species [18], [19]. However, in wild-caught flying foxes, medetomidine alone is not sufficient to produce complete immobilization for the purpose of collecting clinical samples such as blood or oral, rectal, or urogentital swabs. Although 55% of animals induced with medetomidine alone were adequately immobilized and had a quick and uneventful recovery when reversed with atipamezole, 45% of animals were not satisfactorily anesthetized. Incomplete immobilization poses a risk of injury to the handler and increased stress to the animal. If non-invasive procedures are undertaken, such as morphometric measurement, weighing, and banding, medetomidine may be a suitable short-term agent for an experienced field scientist with a competent technician. However, more extensive or invasive sampling (blood collection, throat, rectal, urogenital swabbing, and wing-punch biopsy) that may cause the animal discomfort or stimulate a sedated bat requires more complete anesthesia and immobilization. In this study, stress or excitement from capture and handling may have contributed to the higher anesthetic failure rate associated with the use of medetomidine alone [9], [16]. It should be noted that for the 11 variable flying foxes that were classified as having experienced an anesthetic failure, mechanical or auditory stimulation appeared to decrease the level of sedation achieved when medetomidine was used alone. This is a known effect in dogs and other wildlife sedated with medetomidine [9], [16]. Bats sedated with medetomidine alone also displayed brief excitatory periods prior to sedation as evidenced by vocalization, wing flapping, and biting. Because of concerns of physical injury to the bat or handler, as well as the potential for zoonotic disease transmission via a bite wound, we elected to discontinue the trial of medetomidine alone.

Both doses of medetomidine/ketamine produced complete short-term immobilization in these variable flying foxes, and higher quality immobilization (sufficiently rapid and smooth induction, complete plane of anesthesia, and smooth recovery) compared to medetomidine alone. This allowed for maximal handler and bat safety. When the medetomidine was reversed in bats given either medetomidine/ketamine combination, recovery occasionally involved compulsive movements, including biting, wing-chewing and wing-flapping; this is consistent with adverse effects reported in a variety of species when ketamine alone is used to produce anesthesia [16].

While both dosages of medetomidine/ketamine provided excellent induction and immobilization, the quality and speed of recovery was better for bats receiving the 0.025/2.5 mg/kg dose compared to 0.5/5 mg/kg of medetomidine/ketamine. A trend toward prolonged recovery times at the higher dose of medetomidine/ketamine may be due to a higher dose of ketamine remaining in the system when the medetomidine was partially reversed.

Heard *et. al.* [12] recommended a dose of 0.5/5 mg/kg of medetomidine/ketamine for immobilization for longer than 30 minutes and found that a dose of 0.03/3 mg/kg has a mean immobilization time of 18 minutes. However, in the field most sampling can be conducted within 15 minutes; therefore a lower dose such as the 0.025/2.5 mg/kg dose could be sufficient and would still provide a smooth and fast recovery. While the higher dose of 0.05/k mg/kg medetomidine/ketamine certainly produces a satisfactory anesthetic plane for very short field procedures it may not be the ideal protocol for the type of restraint described in this study, as the use of atipamezole would reverse the medetomidine but the ketamine would remain biologically active and at sufficient levels to likely lead to the spastic movements and prolonged recovery time that our field team noted. Finally, the induction times in our study are longer than those reported by Heard *et. al.*; this most likely because the animals in this study were stressed free-ranging flying foxes that were trapped, whereas the study by Heard *et. al.* used captive animals that were habituated to periodic capture and handling [12]. Although the 0.25/2.5 mg/kg dose had a stable anesthetic plane and quick recovery, a quarter of the animals did require additional doses of anesthetics to be induced, suggesting that a slightly higher dose of 0.3/3 mg/kg medetomidine/ketamine protocol may be more efficient and still minimize the occurrence of prolonged recoveries.

Two pups weighing less than 200 g were immobilized with the 0.5/5 mg/kg dose of medetomidine/ketamine. In both animals, induction was smooth and immobilization was complete. However, recovery of the bat pups was extremely prolonged (9 and 18 minutes) and one became extremely hypothermic (93.8°F). Ultimately, we recommend *not to* chemically immobilize pups given their docile nature, and the ability to safely obtain samples with physical restraint alone.

In conclusion, of the dosages investigated in this study, we recommend a medetomidine/ketamine dose of 0.025/2.5 mg/kg, administered by intravenous injection, for short-term chemical field immobilization of free-ranging variable flying foxes. A higher dose is safe in these bats, however the 0.5/5 mg/kg dose maintains anesthesia for a period longer than necessary for the activities described in this report, often leading to a prolonged recovery when the medetomidine is reversed. We do not recommend the use of medetomidine alone as this protocol failed to produce adequate restraint nearly 50% of the time.

## References

[1] Heard DJ, Fowler ME, Miller RE (2003). Chroptera.. Zoo and Wild Animal Medicine. 5th ed.

[2] Rainey WE, Kunz TH, Racey PA (1998). Conservation of bats on remote Indo-Pacific islands.. Bat Biology and Conservation.

[3] Chua KB, Bellini WJ, Rota PA, Harcourt BH, Tamin A (2000). Nipah virus: A recently emergent deadly paramyxovirus.. Science.

[4] Field H, Young P, Yob JM, Mills J, Hall L (2001). The natural history of Hendra and Nipah viruses.. Microbes and Infection.

[5] McColl KA, Tordo N, Aguilar Sentien AA (2000). Bat lyssavirus infections.. Revue scientifique et technique.

[6] Chua KB, Goh KJ, Wong KT, Kamarulzaman A, Tan PS (1999). Fatal encephalitis due to Nipah virus among pig farmers in Malaysia.. Lancet.

[7] Chua K, Wang LF, Lam SK, Crameri G, Yu M (2001). Tioman virus, a novel paramyxovirus isolated from fruit bats in Malaysia.. Virology.

[8] Chua KB, Lek Koh C, Hooi PS, Wee KF, Khong JH (2002). Isolation of Nipah virus from Malaysian island flying foxes.. Microbes and Infection.

[9] Kreeger TJ (1996). Handbook of Wildlife Chemical Immobilization.

[10] Heard DJ, Beale C, Owens J (1996). Ketamine and ketamine:xylazine ED(50) for short-term immobilization of the island flying fox (Pteropus hypomelanus).. Journal of Zoo and Wildlife Medicine.

[11] Carpenter RE, Fowler ME (1986). Old world fruit bats.. Zoo and Wild Animal Medicine. 2nd ed.

[12] Heard D, Towles J, LeBlanc D (2006). Evaluation of Medetomidine/ketamine for short-term immobilization of variable flying foxes (*Pteropus hypomelanus*).. Journal of Wildlife Diseases.

[13] Jonsson NN, Johnston SD, Field H, de Jong C, Smith C (2004). Field anaesthesia of three Australian species of flying fox.. Veterinary Record.

[14] Sohayati AR, Zaini CM, Hassan L, Epstein J, Suri AS (2008). Ketamine and Xylazine combinations for short-term immobilization of wild variable flying foxes (*Pteropus hypomelanus*).. Journal of Zoo and Wildlife Medicine.

[15] Epstein JH, Prakash V, Smith CS, Daszak P, McLaughlin AB (2008). Henipavirus infection in fruit bats (Pteropus giganteus), India.. Emerging Infectious Diseases.

[16] Plumb DC (2005). Veterinary Drug Handbok.

[17] Breed AC, Field HE, Smith CS, Edmonston J, Meers J (2010). Bats without borders: Long-distance movements and impolications for disease risk management.. EcoHealth.

[18] West G, Heard D, Caulkett N (2007). Zoo Animal and Wildlife Immobilization and Anesthesia.

[19] Jalanka HH, Roeken BO (1990). The use of medetomidine, medetomidine-ketamine combinations, and atipamezole in nondomestic mammals: A review.. Journal of Zoo and Wildlife Medicine.

